# Development and application of classical swine fever virus monoclonal antibodies derived from single B cells

**DOI:** 10.1186/s13567-023-01229-y

**Published:** 2023-10-16

**Authors:** Zhongyuan Ma, Yongcong Zhao, Jianliang Lv, Li Pan

**Affiliations:** State Key Laboratory for Animal Disease Control and Prevention, Gansu Province Research Center for Basic Disciplines of Pathogen Biology, Lanzhou Veterinary Research Institute, Chinese Academy of Agricultural Sciences, Lanzhou, 730046 China

**Keywords:** CSFV, E2, single B cells, epitope, bELISA

## Abstract

Vaccination with E2 subunit vaccines is currently the main measure to control classical swine fever virus (CSFV), which is an endemic disease, and detection of antibodies against CSFV E2 is the most effective way to evaluate herd immunity. In the present study, the E2 protein was expressed by a baculovirus expression system, and two monoclonal antibodies (mAbs), namely, 3A9 and 4F7, were successfully produced using techniques for the isolation of single B cells from splenocytes from mice immunized with the E2 protein. Moreover, two linear B-cell epitopes, ^25^GLTTTWKEYSHDLQL^39^ and ^259^GNTTVKVHASDERGP^273^, reactive to 3A9 and 4F7, respectively, were identified using epitope mapping of the E2 protein. In addition, the diagnostic performance of the two mAbs was evaluated using blocking enzyme-linked immunosorbent assay (bELISA), and the results showed that the two mAbs had high diagnostic specificity (96.08%, 94.38%) and diagnostic sensitivity (97.49%, 95.97%). Together, these findings identify two ideal candidate peptides and matching mAbs for a new method of CSFV diagnosis, which will contribute to the control and eradication of classical swine fever.

## Introduction

Classical swine fever (CSF), which is caused by classical swine fever virus (CSFV), affects domestic and wild pigs, posing a threat to the pig industry and potentially causing economic losses [1]. Currently, strict epidemiological surveillance and prophylactic vaccination remain crucial for the control of CSF, which is an endemic disease [2]. CSFV shares high structural and antigenic homology with bovine viral diarrhoea virus (BVDV) and border disease virus (BDV). In addition, the use of live attenuated vaccines interferes with serological diagnosis because vaccinated animals cannot be easily distinguished from infected animals [3, 4]. Therefore, the development of E2 subunit vaccines and matching diagnostic tools is needed.

CSFV is a positive-sense, single-stranded RNA virus belonging to the family *Flaviviridae* and genus *Pestivirus*. The genome of CSFV contains a single open reading frame (ORF) encoding four structural proteins (SPs: C, E^rns^, E1 and E2) and eight nonstructural proteins (NSPs: N^pro^, P7, NS2, NS3, NS4A, NS4B, NS5A and NS5B). Among these SPs, Erns has been shown to possess endonuclease activity and inhibit early-stage immunization in the host [5, 6]. E2 is the major protective antigen, and it is located on the viral envelope and has numerous antigenic determinants [7]. Thus, monoclonal antibodies (mAbs) directed against the E2 glycoprotein are useful tools for identifying epitopes, which play important roles in vaccine design and diagnosis.

Monoclonal antibodies against CSFV E2 selected from mouse hybridomas have been extensively used to investigate the antigenic profile of CSFV [8]. To date, four antigenic domains (A, B, C, and D) of E2 have been identified in its N-terminal half [9, 10]; these domains constitute two antigenic units, namely, B/C (aa 1–90) and D/A (aa 91–170). Among those, the ^829^TAVSPTTLR^837^ linear epitope in domain A is an important neutralizing epitope recognized by the WH303 mAb, which is commonly used for the evaluation of vaccination and design of new vaccines [11, 12]. However, the inherent characteristics of hybridomas, such as the low fusion rate and ease of chromosome loss, limit the application of mAbs to some extent [13, 14]. With the advent of single-cell sequencing technologies, the amplification of immunoglobulin gene (Ig) fragments from single B cells by utilizing nested PCR has allowed many of these limitations to be bypassed [15, 16], and this technology has been shown to be a versatile tool for the generation of more mAbs [17].

In the present study, we isolated the 3A9 and 4F7 mABs from single B cells of vaccinated mice using biotinylated E2 protein and fluorescein isothiocyanate (FITC)-labelled rat anti-mouse IgM via fluorescence-activated cell sorting (FACS) [18]. Moreover, two linear B-cell epitopes, namely, ^25^GLTTTWKEYSHDLQL^39^ and ^259^GNTTVKVHASDERGP^273^, recognized by 3A9 and 4F7, respectively, were identified using epitope mapping of the E2 glycoprotein. Based on these materials, blocking enzyme-linked immunosorbent assays for detecting antibodies against CSFV E2 were developed, and the diagnostic performance of the two mAbs was evaluated. The results showed that the two mAbs had high signal-to-noise ratios, diagnostic specificities, and diagnostic sensitivities. Together, these findings indicated that the murine 3A9 and 4F7 mAbs isolated from single B cells show potential as candidates for diagnostic reagents.

## Materials and methods

### Serum samples

#### Serum samples from naïve animals

Serum samples from clinically healthy and unvaccinated pigs (*n* = 80) were collected and tested using IDEXX® CSFV bELISA kits (all samples had negative results; blocking rate < 30%). These samples were used to assess diagnostic specificity (Dp).

#### Serum samples from animals vaccinated with the E2 subunit vaccine

E2-positive serum samples (*n* = 160) were collected from pigs that had been inoculated with the E2 subunit vaccine (all samples with positive results had been confirmed using commercial ELISA kits; blocking rate > 40% and levels ranging from low to high). These samples were used to assess diagnostic sensitivity (Dn).

#### Serum samples from pigs inoculated with the conventional live C-strain vaccine

Serum samples (*n* = 30) from pigs inoculated with the conventional live C-strain vaccine were collected 28–42 days post-vaccination by our research group. The samples with positive results were confirmed using IDEXX® CSFV bELISA kits.

#### Standard control sera

CSFV E2 positive control and negative control sera were prepared by our group [19]. A standard positive control (P) and negative control (N) were prepared as internal controls.

### Expression and purification of the CSFV E2 glycoprotein

The coding region of the CSFV E2 gene was synthesized (Nanjing GenScript Biotech Co., Ltd) with the addition of a 5′ terminal signal peptide (sequence: MLLVNQSHQGFNKEHTSKMVSAIVLYVLLAAAAHSAFA) for expression (Bac-to-Bac™ Baculovirus Vector System, Invitrogen) and a downstream linker encoding the sequence GSGS and a hexahistidine tag for protein purification by affinity chromatography. In brief, the E2 gene was cloned and inserted into pFastBac1 (Invitrogen) using the EcoRI and XhoI restriction enzymes according to the manufacturer’s instructions (Bac-to-Bac™ Baculovirus Expression Systems, Invitrogen). The construct was confirmed by digestion and sequencing. The pFastBac1 vector carrying the E2 gene was transformed into *E. coli* DH10Bac competent cells to regenerate bacmid-expressing E2. Colonies containing bacmid DNA were subjected to PCR to confirm transformation. Bacmid DNA (1 µg) containing the E2 gene was transfected into SF9 cells (2 × 10^6^ cells/mL) using 8 µL of Cellfectin transfection reagent (Invitrogen). Transfected cells were grown in 100 mL of Sf-900 II SFM insect cell culture medium (Gibco®, Life Technologies) at 27 °C in an orbital shaker at 135 rpm until the cells showed visible signs of infection. On Day 3 post-transfection, culture supernatants were harvested by centrifugation at 3500 × *g* for 15 min and then filter-sterilized (0.45 μm filter). CSFV E2 protein was purified from the culture supernatant by Ni-chelating SFF affinity chromatography (GE Healthcare) according to the manufacturer’s instructions. The protein eluate was dialyzed against 50 mM Tris and 150 mM NaCl (pH 8.0) and then evaluated by SDS-PAGE and Western blot analysis.

### Biotinylation of E2 protein

Highly purified E2 protein was biotinylated with EZ-Link Sulfo-NHS-LC-biotin reagent (Thermo Fisher Scientific, USA) according to the manufacturer’s instructions, and the resulting biotinylated E2 was named biotin-E2 and evaluated by Western blot analysis.

### Mouse vaccination and single B-cell sorting by FACS

Four- to six-week-old BALB/c mice were inoculated subcutaneously with 25 µg of purified E2 protein in Freund’s adjuvant at 2-week intervals for a total of two inoculations. Splenocytes and blood for serum analysis were harvested from animals 7 days following the final boost vaccination. For staining, freshly isolated splenocytes were first blocked using mouse Fc block reagent (Miltenyi Biotec) according to the manufacturer’s instructions. Next, B cells were stained with biotin-E2 and rat anti-mouse IgM-FITC (Invitrogen, labelled with FITC) for 30 min at 4 °C in PBS buffer containing 2 mM EDTA and 0.5% BSA. The mouse anti-biotin APC secondary antibody (Miltenyi Biotec) was then added, followed by incubation for 20 min at 4 °C. The stained samples were immediately sorted by flow cytometry (BD FACS Aria II, USA) using a 70 μm nozzle. E2-specific plasmablasts were sorted for CSFV E2-APC^+^ and IgM-FITC^−^ events. Before single-cell sorting, 10 µL/well of single-cell lysis buffer containing 1 µL of DNase (Thermo Fisher Scientific, USA) was added into full skirt 96-well plates (Brand, Germany), and the targeted single cells were then sorted at one cell per well by a BD FACSAria FusionTM cell sorter. Immediately, 10 µL/well SuperScriptTM VILOTM Master Mix (Thermo Fisher Scientific, USA) containing random primers was added for the synthesis of cDNA. After 60 min of incubation at 42 °C, the reaction was terminated at 85 °C for 5 min. The cDNA templates were stored at −20 °C for subsequent PCR amplification.

### Amplification, sequencing, and expression of mAbs

Before PCR amplification, the 5′ end sequence of the immunoglobulin (Ig) variable region (gamma chain, lambda chain, and kappa chain) was analysed using a SMARTer® RACE 5′/3′ kit (Takara Biotech) according to the manufacturer’s instructions, and constant region reverse primers were designed by examining published Ig gene segment nucleotide sequences from IMGT®. The identified 5′ forward primers were designed to anneal to the 5′ end of the framework 1 regions in the V-gene segments. The sequences of the 3′ reverse outer primers were located in the constant region with those of the 3′ inner primers for the 2nd round of PCR closer to those of the J genes than to those of the 3′ outer primers for the first round of PCR. The PCR primers are listed in Tables 1, 2 and 3. For the amplification of antibody variable region genes, two seminested PCR amplifications were run per well for the light and heavy chains. All PCRs were performed in 96-well plates in a total volume of 25 µL per well containing 200 nM each primer or total primer mix, 300 µM each dNTP (Invitrogen), and 1.2 U HotStar Taq DNA Polymerase (QIAGEN). The thermocycling program for the first round of PCR was as follows: 94 °C for 15 min followed by 35 cycles at 94 °C for 30 s, 56 °C (Ig gamma chain), 50 °C (Ig kappa chain) or 58 °C (Ig lambda chain) for 30 s, and 72 °C for 55 s; and a final incubation step at 72 °C for 10 min. Seminested or nested second-round PCR was performed with 3.5 µL of the unpurified first-round PCR product using the following thermocycling program: 94 °C for 15 min followed by 50 cycles at 94 °C for 30 s, 60 °C (Ig gamma chain), 55 °C (Ig kappa chain) or 58 °C (Ig lambda chain) for 30 s, and 72 °C for 45 s; and a final incubation step at 72 °C for 10 min. Furthermore, the full-length sequences of the heavy and light chains were further determined using a SMARTer® RACE 5′/3′ kit, and the paired heavy and light chain variable domain genes were then synthesized by GenScript Corporation with codon optimization for expression in HEK293 cells and inserted into the pCDNA3.1 expression vector.

**Table 1:** **Primers used for PCR amplification of the gamma chain of murine IgG**

Primers	Primer sequence^a^ (5′ → 3′)
Igγ forward	AGAGTGCTGATTCTTTTGTG GAATGGCCTTATATCTTTCTC GAATGGAGTTGGATATTTCTC TCCTTGAATCTTTCTCTTCCT GGATTCAGCAGGATCTTTCTC GGGACACAGGACCTCACCAT GGATGGAGCTGTATCATCCTC GGATGGAGCTGTTTCATCGTC GGATTGCTGTTTCTTTTCTTC GAATGGCTGTGGAACTTGCT
Igγ reverse outer	GAAGGTGYGCACACYGCTGGAC (1st PCR)
Igγ reverse inner	GCTCAGGGAARTAGCCCTTGAC (2nd PCR)

^a^Degenerate bases, including S = C or G, Y = C or T, and R = A or G, were synthesized in these sequences.

**Table 2:** **Primers used for PCR amplification of the lambda chain of murine IgG**

Primers	Primer sequence^a^ (5′ → 3′)
Igλ forward	GAGACAGACACAATCCTGCTA GATTTTCAAGTGCAGATTTTC AAGTTGCCTGTTAGGCTGTTG GATTCACAGGCCCAGGTTCTT GAATCACAGACTCAGGTCCTC TTACTGCTGCTATGGGTATCT ACCATGTTCTCACTAGCTCTT TTCTGGATTCCTGCTTCCAGC AGGGTCCTTGCTGAGCTCCTG GAGACAGACACACTCCTGTTA GAGACAGACACACTCCTGCTA ACCATGCTCTCACTAGCTCCT
Igλ reverse outer	GTACCATYTGCCTTCCAGKCCACT (1st PCR)
Igλ reverse inner	CTCYTCAGRGGAAGGTGGRAACA (2nd PCR)

^a^Degenerate bases, including S = C or G, Y = C or T, and R = A or G, were synthesized in these sequences.

**Table 3:** **Primers used for PCR amplification of the kappa chain of murine IgG**

Primers	Primer sequence^a^ (5′ → 3′)
Igκ forward	GCTGTTTTGTATACCTGGG CTCAGCTCCTGTTGCTGTGGC GTGTMTGGTGCTBRTGGG GGGTATCTGGTACCTGTGG CCTCATATTTTTGCTGCTATGGG TGCTTTTCTGGATTTCAGCCTCC TCAACTTCTGCTCTTCCTGCTGT CTAGCTCYTCTCCTCAGYCTTCTT
Igκ reverse outer	ACTGAGGCACCTCCAGATG (1st PCR)
Igκ reverse inner	TGGGAAGATGGATACAGTT (2nd PCR)

^a^Degenerate bases, including S = C or G, Y = C or T, and R = A or G, were synthesized in these sequences.

### Preparation of HRP-conjugated mAbs

After the immunoreactivity of the mAbs was confirmed by Western blot analysis, the expressed and purified mAbs mentioned above were labelled with HRP using the Lightning-link® HRP Conjugation Kit (Expedeon), and the HRP-conjugated mAbs were stored at −20 °C until use.

### Epitope mapping

To map linear epitopes, overlapping peptides of E2 (15 amino acids in length, overlapping each other by 10 amino acids) were synthesized by Sangon Biotech (Shanghai, China) based on the E2 protein sequence (residues 1–342 without the transmembrane region) derived from the Shimen strain (GenBank number AAK21202.1). All peptides were screened with mAbs by indirect ELISA. In brief, the synthesized peptides were coated and identified using a peptide coating kit (Takara, MK100) according to the manufacturer’s instructions.

### Immunofluorescence assay (IFA)

Porcine kidney (PK-15) cells (ATCC® CCL-33™) seeded in plates (Corning, NY, USA) were infected with the CSFV C-strain. Forty-eight hours post-infection, the cells were fixed with 80% ice-cold acetone in PBS for 30 min at −20 °C and washed with PBS twice. After blocking with 5% (w/v) nonfat milk in PBS and washing twice with PBS, the cells were incubated with different primary antibodies for 1 h at 37 °C. After rinsing with PBS twice, a 1:2000 dilution of the mAbs (3A9 and 4F7) and a FITC-conjugated goat-anti-mouse antibody (Thermo Scientific, USA) was added to each well for a 1 h incubation at 37 °C. Following three washes, the signal was visualized with a confocal laser scanning microscope (Leica, Wetzlar, Germany).

### Measurement of mAb concentrations using blocking ELISA

The wells of ELISA plates (Costar, catalogue number: 42592) were coated with 100 µL of synthesized peptides (200 ng/well, 100 ng/well, 50 ng/well, and 25 ng/well) diluted in PBS buffer (pH 7.4) at 4 °C overnight. The plates were then thoroughly washed with PBST (PBS containing 0.05% Tween 20) and blocked with PBST containing 1% BSA and 5% sucrose at 37 °C for 2 h. After five washes with PBST, positive and negative control serum samples were diluted (1:2, 1:4, 1:8, and 1:16) in the abovementioned sample diluent. The diluted sera were transferred to the coated plates (100 µL per well), and the plates were incubated at 37 °C for 60 min. After five washes, 100 µL of HRP-conjugated monoclonal antibodies (1:20 000 dilution) was added to each well prior to incubation for 30 min at 37 °C. After washing, colour was developed with 100 µL of TMB substrate at 37 °C for 10 min, and the reaction was terminated with 100 µL of 2 M H_2_SO_4_. Finally, the absorbance at 450 nm (OD450) was measured using a Varioskan Lux instrument. After the optimum coating concentration and serum dilution were confirmed, swine serum samples with a known status were tested to evaluate the diagnostic performance of the mAbs. The sample results were recorded as percent inhibition (PI) values calculated using the following formula: PI = (OD450neg − OD450sample) × 100%/(OD450neg − OD450pos). The OD450 values were also recorded as percent inhibition (PI) values calculated using the same formula. All PI values of the assay were used to analyse diagnostic specificity and diagnostic sensitivity using MedCalc software.

## Results

### Production and biotinylation of E2 protein

**Figure 1:**
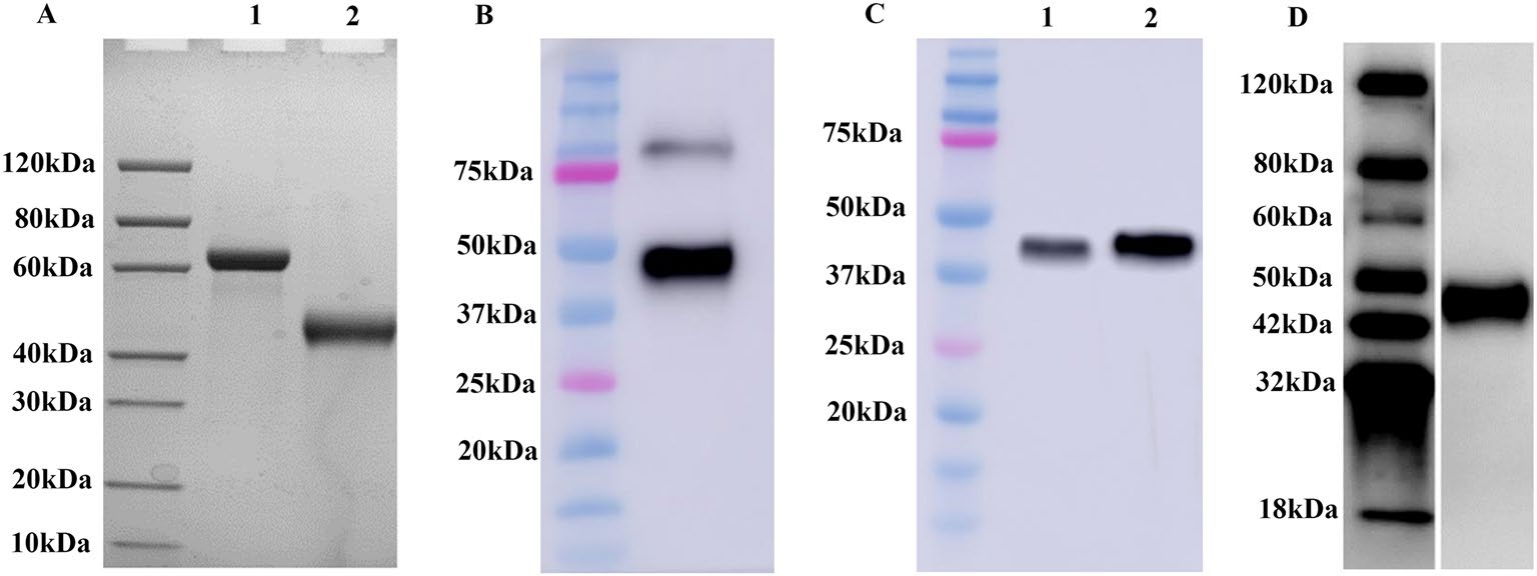
**SDS-PAGE and Western blot analysis of CSFV E2. A** Recombinant E2 proteins were expressed using SF9 insect cells, and the molecular weight of the expressed E2 protein was 43 kDa. Lane 1, BSA (2 µg); Lane 2, purified E2 (reducing conditions, 2 µg). **B** Purified E2 protein was analysed by Western blotting using a mouse anti-His mAb (GenScript), and dimer bands were observed. **C** Highly purified E2 protein was biotinylated with EZ-Link Sulfo-NHS-LC-biotin reagent, and biotinylated E2 was recognized by streptavidin-HRP (GenScript). Lane 1, 0.1 µg; Lane 2, 0.2 µg. **D** Purified E2 protein was further analysed by Western blotting using CSFV-positive serum, and the expressed E2 protein was accurately recognized by specific antibodies.

The CSFV E2 gene from the CSFV Shimen strain was cloned and inserted into a recombinant baculoviral vector, and recombinant proteins were expressed using SF9 insect cells. The culture medium was then collected by centrifugation. The supernatant was subjected to protein purification with Ni-NTA agarose beads. The expression of soluble His-tagged E2 protein was confirmed by SDS-PAGE. As shown in Figure 1A, the expressed E2 protein had a molecular weight of 43 kDa, and the signal peptide was excluded. Dimer bands were also observed in the Western blot analysis of the E2 protein (Figure 1B). Moreover, the proteins ran at a higher molecular weight than the predicted mass calculated from the amino acid composition, indicating that the two proteins were glycosylated. Highly purified E2 protein was biotinylated with EZ-Link Sulfo-NHS-LC-biotin reagent, and the resulting biotinylated E2 was named biotin-E2 and evaluated by Western blot analysis. As shown in Figure 1C, biotin-E2 reacted with streptavidin-HRP. Furthermore, the immunoreactivity of purified E2 protein was tested using standard positive control serum by Western blot analysis. As shown in Figure 1D, the results showed that positive serum produced a strong signal corresponding to the E2 protein.

### Isolation of single B cells from vaccinated mice and mAb generation

**Figure 2:**
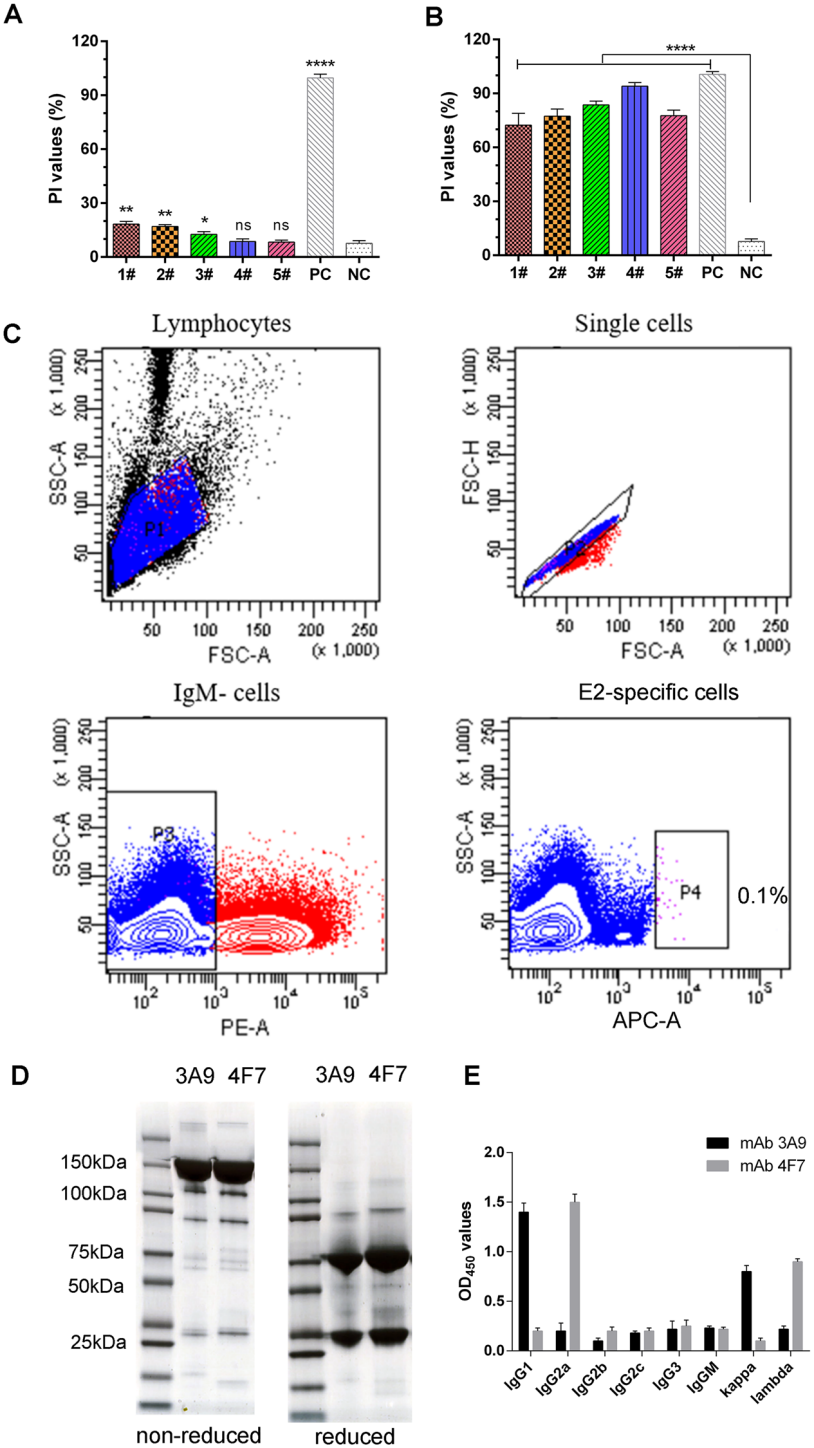
**Isolation of single B cells and generation of mAbs against CSFV. A** Anti-CSFV antibodies were detected using a CSFV antibody test kit (positive blocking rate ≥ 40% and negative blocking rate ≤ 30%). PI values of serum samples isolated from unvaccinated mice. **B** PI values of serum samples isolated from individual mice immunized with the E2 subunit vaccine. The results were obtained from at least three biological replicates (mean ± SD) and analysed using a t test with GraphPad Prism software; ns, not statistically significant; *p < 0.05, **p < 0.005 and ****p < 0.0001 (versus the negative control, NC). **C** Isolation of single B cells from vaccinated mice via FACS. Single B cells from the splenocyte population of mouse no. 4 were used to isolate single B cells stained with biotin-E2-APC and rat anti-mouse IgM-FITC. CSFV E2-specific plasmablasts isolated from splenocytes in P4 constituted approximately 0.1% of the cell population. **D** The mAbs 3A9 and 4F7 were successfully expressed in HEK293 cells, as shown by nonreducing and reducing SDS-PAGE. **E** mAb isotyping was performed according to the manufacturer’s instructions. The result was based on measurement of the OD at 450 nm.

Individual mice were immunized with an E2 subunit vaccine to generate a spectrum of antibody responses with high levels of blocking, which was evaluated using a Classical Swine Fever Virus Antibody Test Kit (IDEXX). Each immunized mouse exhibited a robust antibody response (Figures 2A, B). As shown in Figure 2C, splenocytes from mouse no. 4 were used to isolate single B cells that were stained with biotin-E2-APC and rat anti-mouse IgM-FITC by FACS. CSFV E2-specific plasmablasts isolated from splenocytes constituted approximately 0.1% of the cell population. All monoclonal antibodies were obtained from 100 single E2-specific IgG^+^ B cells. Among these single clones, 3A9 and 4F7 were successfully expressed in HEK293 cells and then purified, as shown by nonreducing and reducing SDS-PAGE (Figure 2D). Using a commercial isotype classification kit, mAb 3A9 was identified to be in the IgG1 subclass, with κ-type light chains, and mAb 4F7 was identified to be in the IgG2a subclass, with λ-type light chains (Figure 2E).

### Epitope mapping of the E2 protein with the 3A9 and 4F7 mAbs

**Figure 3:**
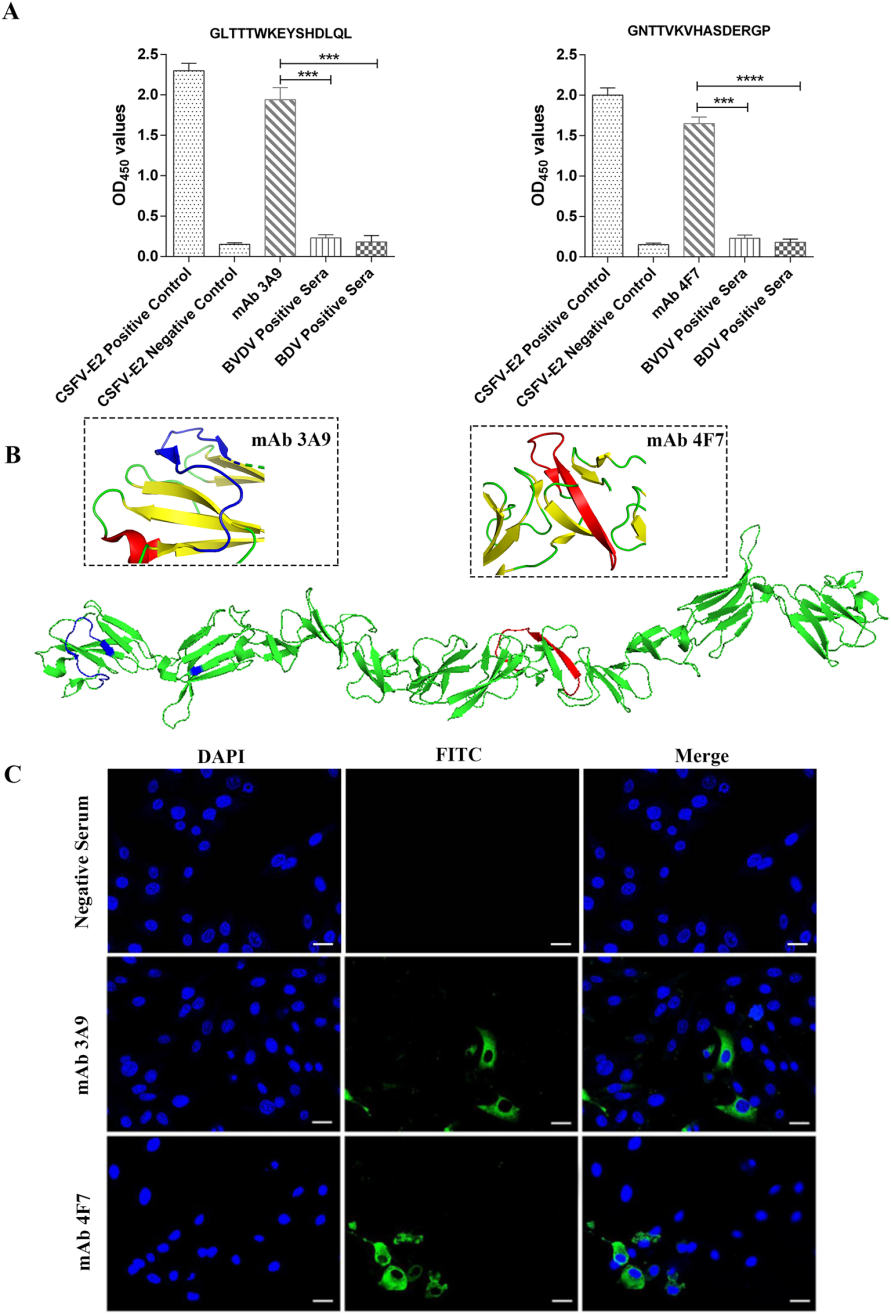
**Identification of linear epitopes and serological detection.** **A** Indirect ELISAs were performed using synthetic peptides to verify the reactivity of the 3A9 and 4F7 mAbs. The inset shows that the 3A9 mAb can react with ^25^GLTTTWKEYSHDLQL^39^ and that the 4F7 mAb can react with ^259^GNTTVKVHASDERGP^273^. Neither epitope was cross-reactive with sera positive for BVDV or BDV. The results were obtained from at least three biological replicates (mean ± SD) and analysed using a t test with GraphPad Prism software; ***p < 0.001 and ****p < 0.0001. Characteristic analysis of the linear B-cell epitope of the VP2 protein. **B** The relative spatial position of the identified epitope is presented in a surface view from a partially predicted 3D structure of CSFV E2 (reference structure, PDB ID: 2YQ2). The ^25^GLTTTWKEYSHDLQL^39^ linear epitope recognized by the 3A9 mAb is shown in blue, and the ^259^GNTTVKVHASDERGP^273^ epitope recognized by the 4F7 mAb is shown in red. **C** Indirect immunofluorescence analysis of the immunoreactivity of the mAbs. PK-15 cells were used for indirect immunofluorescence assays with the 3A9 and 4F7 mAbs (green), and nuclei were stained with DAPI (blue). Fluorescence images were acquired with a confocal laser scanning microscope. Bright field, scale bar = 25 μm.

To define major epitopes on the CSFV E2 protein, peptides representing the whole polypeptide of E2 were synthesized and then analysed by indirect ELISA. As shown in Figure 3A, the identified ^25^GLTTTWKEYSHDLQL^39^ linear epitope was recognized by the 3A9 mAb, while the ^259^GNTTVKVHASDERGP^273^ linear epitope reacted with the 4F7 mAb. Both mAbs exhibited high OD values and showed high binding affinity for the CSFV E2 protein. Indirect ELISAs also showed that the two identified epitopes were not cross-reactive with serum samples positive for the BVDV and BDV strains. To understand the structural mechanism of the epitope identified in the mAbs, the X-ray crystal structure of BVDV E2 (PDB ID: 2YQ2) was used as the reference structure, and the peptide was analysed using the computer software program PyMOL2.5. The results revealed that the two linear epitopes were exposed on the surface of the predicted E2 protein (Figure 3B). In addition, IFA showed that both the 3A9 and 4F7 mAbs successfully detected CSFV E2 in PK-15 cells but no significant reactivity to serum from unimmunized mice (Figure 3C). Together, these findings showed that the two ideal candidate peptides and the matching mAbs can be used for the diagnosis of CSFV.

### Construction and standardization of blocking ELISAs

**Figure 4:**
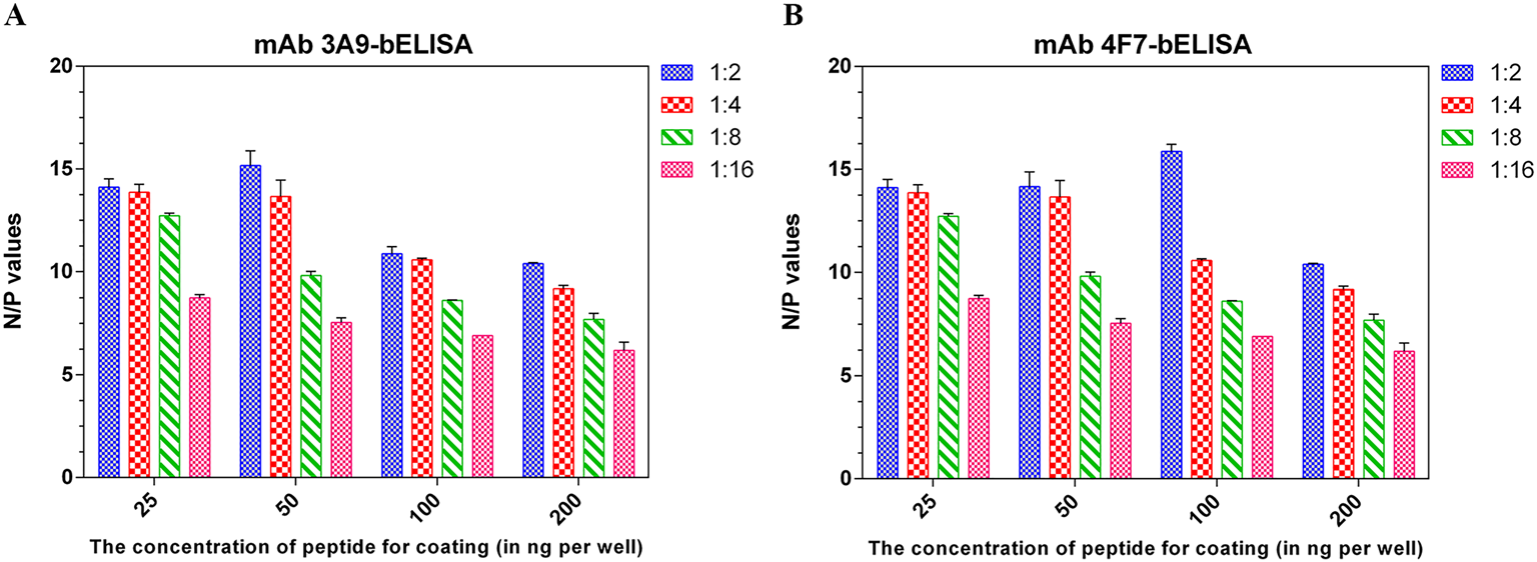
**Results of checkerboard titration assays used to optimize the peptide concentration and serum dilution.** Standard positive (P) and standard negative (N) serum samples were serially diluted twofold, and the coating concentration was varied. **A** For the mAb 3A9 bELISA, the optimal conditions were a peptide coating concentration of 50 ng/well and a serum dilution ratio of 1:2. Under the optimal conditions, the best signal-to-noise ratio (N/P) was 15. **B** For the mAb 4F7 bELISA, the optimal conditions were a peptide coating concentration of 100 ng/well and a serum dilution ratio of 1:2. Under the optimal conditions, the best signal-to-noise ratio (N/P) was 16.

The optimum peptide coating concentration and serum dilution were determined using a checkerboard titration method. For mAb 3A9-based bELISA (mAb 3A9-bELISA), the concentration of the linear epitope ^25^GLTTTWKEYSHDLQL^39^ and the test serum dilution were fixed at 50 ng/well and 1:2, respectively. For mAb 4F7-based bELISA (mAb 4F7-bELISA), the concentration of the linear epitope ^259^GNTTVKV-HASDERGP^273^ and the test serum dilution were fixed at 100 ng/well and 1:2, respectively. As shown in Figures 4A, B, under the optimal conditions, both bELISAs exhibited a high signal-to-noise ratio (N/P value).

### Both 3A9 and 4F7 exhibit high diagnostic sensitivity and specificity

**Figure 5:**
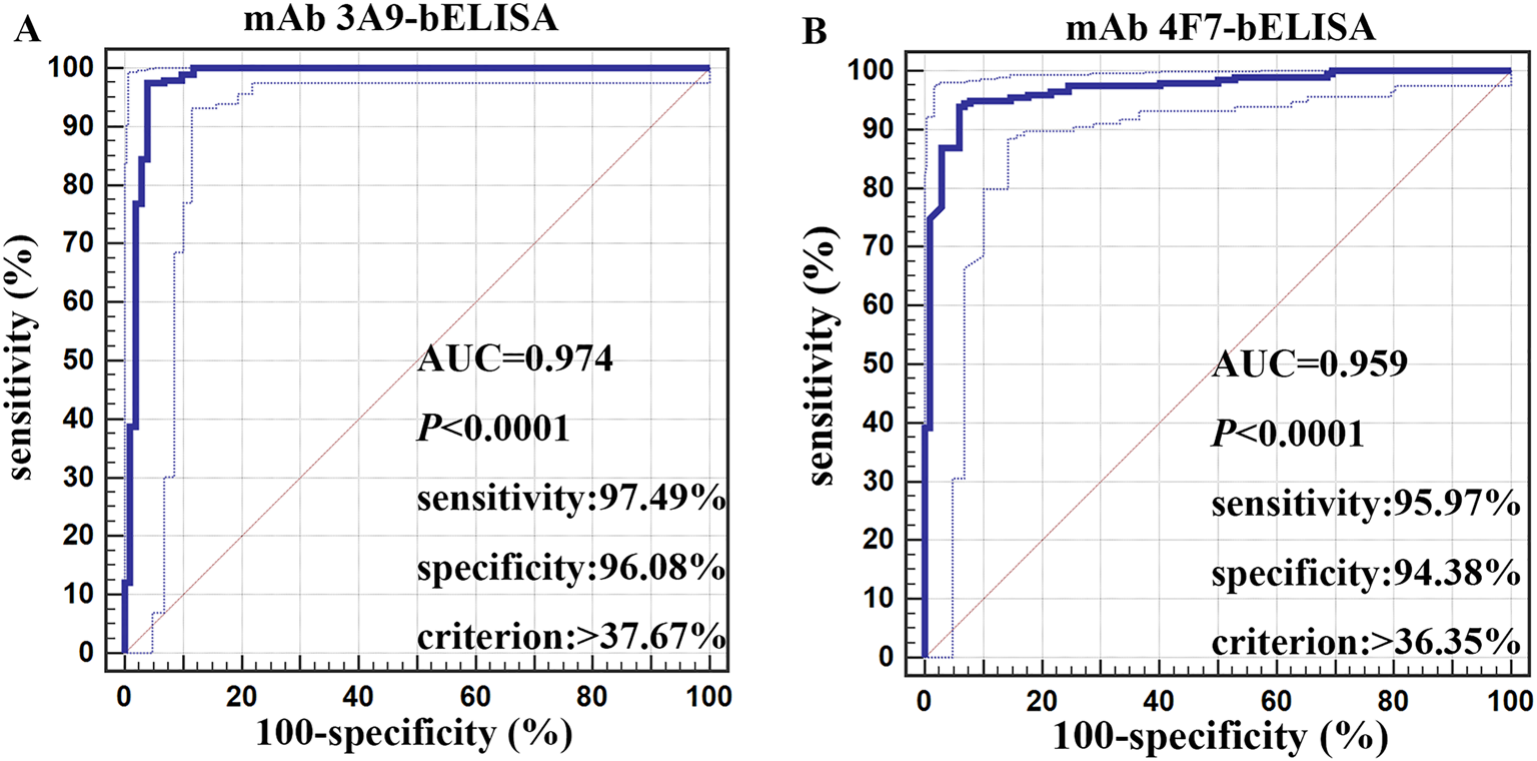
**Estimation and comparison of the Dn and Dp values in the two assays by receiver operating characteristic (ROC) curve analysis.** Each point on the ROC curve represents a sensitivity-specificity pair associated with a particular threshold. **A** For the 3A9 mAb bELISA, the optimal Dn and Dp values were 97.49% and 96.08%, respectively, and the cut-off value was 37.67%. **B** For the 4F7 mAb bELISA, the optimal Dn and Dp values were 95.97% and 94.38%, respectively, and the cut-off value was 36.35%.

A total of 270 serum samples (30 serum samples from naïve animals, 160 serum samples from animals vaccinated with the E2 subunit vaccine, and 30 serum samples from animals vaccinated with the conventional live C-strain vaccine) with known status were used to estimate the Dn and Dp of the 3A9 and 4F7 mAbs by receiver operating characteristic (ROC) curve analysis (Figures 5A, B). According to ROC curve analysis, the sensitivity and specificity values of the 3A9 mAb were optimal when the cut-off value was 37.67%, and the Dn and Dp were 97.49% and 96.08%, respectively. The sensitivity and specificity values of the 4F7 mAb were optimal when the cut-off value was 36.35%, and the Dn and Dp were 95.97% and 94.38%, respectively. In addition, cross-reactivity with BVDV and BDV was evaluated using four serum samples positive for BVDV and nine serum samples positive for BVDV. According to the criteria of the bELISAs, the PI values of all 13 serum samples were lower than 30%. Thus, the serum samples positive for BVDV and BDV were considered negative.

## Discussion

CSF is a severe problem in the swine industry, primarily in endemic countries and in areas with a wild boar reservoir [20]. Vaccination and surveillance programs are currently the main measures used to control CSF. CSFV shares high structural and antigenic homology with bovine viral diarrhoea virus (BVDV) and border disease virus (BDV), and the use of live attenuated vaccines interferes with serological diagnosis because vaccinated animals cannot be easily distinguished from infected animals [21]. To address these issues, recent studies have aimed to design marked vaccines based on the E2 protein to develop new diagnostic tools [22].

CSFV glycoprotein E2 is one of the most important immunogenic proteins [23], and the glycoprotein Erns of CSFV is an additional antigen that can be involved in protection against CSFV infection [24]. Both E2 and Erns are targets of neutralizing antibodies and are involved in immune protection against CSFV. In the present study, the E2 protein was expressed using SF9 insect cells. Two mAbs, namely, 3A9 and 4F7, were successfully produced using a combination of FACS and techniques for the isolation of single B cells from mice immunized with the E2 protein. Compared to mAbs traditionally prepared from hybridomas, the amplification of immunoglobulin gene (Ig) fragments from single B cells by utilizing nested PCR overcomes traditional limitations, such as low fusion rates and stability issues [25]. In addition, recombinant mAbs can be produced in large amounts to provide sufficient material for various applications.

To map linear epitopes, overlapping peptides of the CSFV E2 protein (overlapping each other by 10 amino acids) were synthesized. Among these peptides, the ^25^GLTTTWKEYSHDLQL^39^ linear epitope reacted with the 3A9 mAb, while the ^259^GNTTVKVHASDERGP^273^ linear epitope reacted with the 4F7 mAb. Both mAbs from single B cells showed high binding affinity for the CSFV E2 protein. In addition, indirect ELISAs showed that the two epitopes were not cross-reactive with serum samples positive for BVDV and BDV.

In conclusion, the present study describes an efficient method for the generation of monoclonal antibodies derived from single B cells. These monoclonal antibodies can be applied to identify conserved epitopes of CSFV E2 and to provide further insights for the design of CSFV vaccines. Based on these materials, blocking ELISAs for detecting antibodies against CSFV E2 were also developed, and the diagnostic performance of the two mAbs was evaluated. The present results showed that the two mAbs had high diagnostic specificity and diagnostic sensitivity. Together, these findings indicated that the two linear epitopes as well as the murine 3A9 and 4F7 mAbs isolated from single B cells show potential as candidates for diagnostic reagents.

## Data Availability

The sequences of the CSFV mAbs described in this study are available from the corresponding author by request.

## References

[1] Reimann I, Depner K, Trapp S, Beer M (2004). An avirulent chimeric pestivirus with altered cell tropism protects pigs against lethal infection with classical swine fever virus. Virology.

[2] Bouma A, De Smit AJ, De Jong MC, De Kluijver EP, Moormann RJ (2000). Determination of the onset of the herd-immunity induced by the E2 sub-unit vaccine against classical swine fever virus. Vaccine.

[3] Luo Y, Li S, Sun Y, Qiu HJ (2014). Classical swine fever in China: a minireview. Vet Microbiol.

[4] Van Rijn PA (2007). A common neutralizing epitope on envelope glycoprotein E2 of different pestiviruses: implications for improvement of vaccines and diagnostics for classical swine fever (CSF)?. Vet Microbiol.

[5] Ji W, Guo Z, Ding NZ, He CQ (2015). Studying classical swine fever virus: making the best of a bad virus. Virus Res.

[6] Zhang H, Wen W, Zhao Z, Wang J, Chen H, Qian P, Li X (2018). Enhanced protective immunity to CSFV E2 subunit vaccine by using IFN-gamma as immunoadjuvant in weaning piglets. Vaccine.

[7] Ganges L, Nunez JI, Sobrino F, Borrego B, Fernandez-Borges N, Frias-Lepoureau MT, Rodriguez F (2018). Recent advances in the development of recombinant vaccines against classical swine fever virus: cellular responses also play a role in protection. Vet J.

[8] Cheng CY, Wu CW, Lin GJ, Lee WC, Chien MS, Huang C (2014). Enhancing expression of the classical swine fever virus glycoprotein E2 in yeast and its application to a blocking ELISA. J Biotechnol.

[9] Chang CY, Huang CC, Deng MC, Huang YL, Lin YJ, Liu HM, Lin YL, Wang FI (2012). Identification of conformational epitopes and antigen-specific residues at the D/A domains and the extramembrane C-terminal region of E2 glycoprotein of classical swine fever virus. Virus Res.

[10] Chang CY, Huang CC, Lin YJ, Deng MC, Tsai CH, Chang WM, Wang FI (2010). Identification of antigen-specific residues on E2 glycoprotein of classical swine fever virus. Virus Res.

[11] Dong XN, Qi Y, Ying J, Chen X, Chen YH (2006). Candidate peptide-vaccine induced potent protection against CSFV and identified a principal sequential neutralizing determinant on E2. Vaccine.

[12] Xu H, Han G, Lu Y, Liu Z, Tao L, He F (2021). Broad neutralization of CSFV with novel monoclonal antibodies in vivo. Int J Biol Macromol.

[13] Pasqualini R, Arap W (2004). Hybridoma-free generation of monoclonal antibodies. Proc Natl Acad Sci USA.

[14] Reichert JM, Rosensweig CJ, Faden LB, Dewitz MC (2005). Monoclonal antibody successes in the clinic. Nat Biotechnol.

[15] Von Boehmer L, Liu C, Ackerman S, Gitlin AD, Wang Q, Gazumyan A, Nussenzweig MC (2016). Sequencing and cloning of antigen-specific antibodies from mouse memory B cells. Nat Protoc.

[16] Wilson PC, Andrews SF (2012). Tools to therapeutically harness the human antibody response. Nat Rev Immunol.

[17] Dong H, Su A, Lv D, Ma L, Dong J, Guo N, Ren L, Jiao H, Pang D, Ouyan H (2019). Development of whole-porcine monoclonal antibodies with potent neutralization activity against classical swine fever virus from single B cells. ACS Synth Biol.

[18] Qi Y, Liu LC, Zhang BQ, Shen Z, Wang J, Chen YH (2008). Characterization of antibody responses against a neutralizing epitope on the glycoprotein E2 of classical swine fever virus. Arch Virol.

[19] Ma Z, Lv J, Zhang Z, Zhao Y, Pan L, Zhang Y (2020). A chemiluminescence immunoassay for rapid detection of classical swine fever virus E2 antibodies in pig serum samples. Transbound Emerg Dis.

[20] Rossi S, Staubach C, Blome S, Guberti V, Thulke HH, Vos A, Koenen F, Le Potier MF (2015). Controlling of CSFV in European wild boar using oral vaccination: a review. Front Microbiol.

[21] Malik YS, Bhat S, Kumar ORV, Yadav AK, Sircar S, Ansari MI, Sarma DK, Rajkhowa TK, Ghosh S, Dhama K (2020). Classical swine fever virus biology, clinicopathology, diagnosis, vaccines and a meta-analysis of prevalence: a review from the indian perspective. Pathogens.

[22] Li GX, Zhou YJ, Yu H, Li L, Wang YX, Tong W, Hou JW, Xu YZ, Zhu JP, Xu AT, Tong GZ (2012). A novel dendrimeric peptide induces high level neutralizing antibodies against classical swine fever virus in rabbits. Vet Microbiol.

[23] Chen JY, Wu CM, Liao CM, Chen KC, You CC, Wang YW, Huang C, Chien MS (2019). The impact of porcine circovirus associated diseases on live attenuated classical swine fever vaccine in field farm applications. Vaccine.

[24] Cheng CY, Wu CW, Chien MS, Huang C (2019). N-terminus of classical swine fever virus strain TD96 glycoprotein E(rns) contains a potential heparin-binding domain. Vet Microbiol.

[25] Cao Y, Li K, Wang S, Fu Y, Sun P, Li P, Bai X, Zhang J, Ma X, Xing X, Zhou S, Bao H, Li D, Chen Y, Li Z, Lu Z, Liu Z (2019). Implication of broadly neutralizing bovine monoclonal antibodies in the development of an enzyme-linked immunosorbent assay for detecting neutralizing antibodies against foot-and-mouth disease virus serotype O. J Clin Microbiol.

